# Supplementary material for: Finding easy regions for short-read variant calling from pangenome data
Source: Gigascience. 2025 Sep 4;14:giaf103. doi: 10.1093/gigascience/giaf103 (PMC12409275; doi:10.1093/gigascience/giaf103)
Supplement: giaf103_GIGA-D-25-00268_Revision_1 [file giaf103_giga-d-25-00268_revision_1.pdf]

GigaScience

Finding easy regions for short-read variant calling from pangenome data

--Manuscript Draft--

**Manuscript Number:**	GIGA-D-25-00268R1
**Full Title:**	Finding easy regions for short-read variant calling from pangenome data
**Article Type:**	Data Note
**Funding Information:**	National Human Genome Research Institute (R01HG010040) — Dr. Heng Li<br>National Human Genome Research Institute (U01HG013748) — Dr. Heng Li<br>National Human Genome Research Institute (U41HG010972) — Dr. Heng Li
**Abstract:**	Background: While benchmarks on short-read variant calling suggest low error rate below 0.5%, they are only applicable to predefined confident regions. For a human sample without such regions, the error rate could be 10 times higher. Although multiple sets of easy regions have been identified to alleviate the issue, they fail to consider non-reference samples or are biased towards existing short-read data or aligners.<br>Results: Here, using hundreds of high-quality human assemblies, we derived a set of sample-agnostic easy regions where short-read variant calling reaches high accuracy. These regions cover 88.2% of GRCh38, 92.2% of coding regions and 96.3% of ClinVar pathogenic variants. They achieve a good balance between coverage and easiness and can be generated for other human assemblies or species with multiple well assembled genomes.<br>Conclusion: This resource provides a convient and powerful way to filter spurious variant calls for clinical or research human samples.
**Corresponding Author:**	Heng Li<br>Dana-Farber Cancer Institute<br>Boston, UNITED STATES
**Corresponding Author Secondary Information:**	
**Corresponding Author's Institution:**	Dana-Farber Cancer Institute
**Corresponding Author's Secondary Institution:**	
**First Author:**	Heng Li
**First Author Secondary Information:**	
**Order of Authors:**	Heng Li
**Order of Authors Secondary Information:**	
**Response to Reviewers:**	Responses to reviewers' comments are uploaded along with the manuscript
**Additional Information:**	
**Question**	**Response**
Are you submitting this manuscript to a special series or article collection?	No
**Experimental design and statistics**<br><br>Full details of the experimental design and statistical methods used should be given	Yes

Powered by Editorial Manager® and ProduXion Manager® from Aries Systems Corporation

in the Methods section, as detailed in our Minimum Standards Reporting Checklist. Information essential to interpreting the data presented should be made available in the figure legends.<br><br>Have you included all the information requested in your manuscript?	
**Resources**<br><br>A description of all resources used, including antibodies, cell lines, animals and software tools, with enough information to allow them to be uniquely identified, should be included in the Methods section. Authors are strongly encouraged to cite Research Resource Identifiers (RRIDs) for antibodies, model organisms and tools, where possible.<br><br>Have you included the information requested as detailed in our Minimum Standards Reporting Checklist?	Yes
**Availability of data and materials**<br><br>All datasets and code on which the conclusions of the paper rely must be either included in your submission or deposited in publicly available repositories (where available and ethically appropriate), referencing such data using a unique identifier in the references and in the “Availability of Data and Materials” section of your manuscript.<br><br>Have you have met the above requirement as detailed in our Minimum Standards Reporting Checklist?	Yes
GigaScience has policies and guidelines in place for the use of generative AI-writing tools such as ChatGPT. If you have used such writing tools to assist with writing the manuscript this must be declared and cited in the text. Authors	No

Powered by Editorial Manager® and ProduXion Manager® from Aries Systems Corporation

should not list AI-writing tools and other AI-assisted technologies as an author or co-author and should acknowledge that they are fully responsible for text generated or refined by AI-writing tools.<p><br><br>A summary of use (particularly in the introduction or among methods) needs to be included at the end of the paper, and the outputs should also be included as a supplementary file hosted in GigaDB or other open repositories. Please read our guidelines for more information. <p><br><br>By submitting to GigaScience, you are aware of the journal's AI-writing tools policy, and if you have declared use of such tools below, you have acknowledged this where appropriate in your manuscript and have made a summary of use and outputs available. <p><br><b>AI-assisted writing tools have been used in the preparation of this manuscript?	

Manuscript

Click here to access/download;LaTeX - Main Document (TeX file);panmask.tex

Placeholder for OUP logo oup.pdf

GigaScience, 2025, 1–4

doi: xx.xxxx/xxxx
Manuscript in Preparation
Data Note

DATA NOTE

Finding easy regions for short-read variant calling from pangenome data

Heng Li[1,2,3,*]

[1]Department of Biomedical Informatics, Harvard Medical School, 10 Shattuck St, Boston, MA 02215, USA and [2]Department of Data Science, Dana-Farber Cancer Institute, 450 Brookline Ave, Boston, MA 02215, USA and [3]Broad Insitute of MIT and Harvard, 415 Main St, Cambridge, MA 02142, USA

[*]hli@ds.dfci.harvard.edu

ORCID: Heng Li [0000-0003-4874-2874]

Abstract

Background: While benchmarks on short-read variant calling suggest low error rate below 0.5%, they are only applicable to predefined confident regions. For a human sample without such regions, the error rate could be 10 times higher. Although multiple sets of easy regions have been identified to alleviate the issue, they fail to consider non-reference samples or are biased towards existing short-read data or aligners.

Results: Here, using hundreds of high-quality human assemblies, we derived a set of sample-agnostic easy regions where short-read variant calling reaches high accuracy. These regions cover 88.2% of GRCh38, 92.2% of coding regions and 96.3% of ClinVar pathogenic variants. They achieve a good balance between coverage and easiness and can be generated for other human assemblies or species with multiple well assembled genomes.

Conclusion: This resource provides a convient and powerful way to filter spurious variant calls for clinical or research human samples.

Key words: variant calling; pangenome; assembly

Introduction

Variant calling from whole-genome sequencing data plays a crucial role in medical genetics, population genetics, and clinical genetic testing. Many algorithms have been developed for accurate variant calling from short-read data [1, 2, 3, 4] and multiple benchmarks have been conducted to evaluate their accuracy [5, 6].

A common practice in variant calling evaluation is to compare called variants to curated ground truth, notably generated by the Genome-In-A-Bottle (GIAB) group [5]. Benchmarks using GIAB samples often report a low error rate of <0.5% [7]. On the other hand, when we compare variant calls from two callers, such as DeepVariant and GATK, their difference often exceeds 5%, 10 times higher than the error rate on GIAB samples. We see this apparent discrepancy because with GIAB, we only evaluate calls in sample-specific *confident regions* where the ground truth can be trusted. The error rate outside confident regions is much higher. The problem is that we do not know confident regions for a clinical sample. If we blindly take calls across the whole genome, the error rate will be closer to 5% than to 0.5%. Arguably, confident regions are more important than variant calling algorithms on GIAB-based benchmarks.

Whereas confident regions are used for variant calling benchmarks on a specific sample, *easy regions* are derived for variant filtration agnostic to samples. They are genomic regions where general variant callers can reach high accuracy without sophisticated filtering. At same time, we also prefer to have large easy regions such that most clinically relevant variants can be retained. Constructing high-quality easy regions demands the balance between genome coverage and ease of calling.

Broadly speaking, there are two classes of methods to identify easy regions. The first only considers unique regions in the refer-

Compiled on: August 15, 2025.
Draft manuscript prepared by the author.

ence genome. Methods in this class differ in how to define uniqueness. We derived a set of easy regions for ancient DNA variant filtering by selecting 35-mers that could not be mapped elsewhere within one mismatch or gap. ENCODE constructed similar k-mer-based sets with GEM mapper [8] and switched to Umap [9] later. GIAB combined short-range uniqueness of GenMap [10] and long-range segmental duplications [11]. It optionally excluded short tandem repeats, regions of excessively low or high GC, and X-transposed and amplicon regions on X and Y chromosomes. Using the reference genome only, methods in this class are fast to run and easy to deploy. However, they would overlook regions unique in the reference genome but duplicated in non-reference samples.

The second class of methods solve this problem by taking non-reference samples into account. We inspect the read alignment of multiple samples and exclude regions of excessively high or low coverage or enriched with alignments of low mapping quality. The 1000 Genomes Project (1KG) was the first to use such an approach [12]. Illumina posted an article in 2021 with a similar method but not citing 1KG. Aganezov et al. [13] reproduced these regions for T2T-CHM13 [14]. Pan et al. [15] went a step further by using multiple aligners and variant callers. ENCODE blacklist [16] considered empirical read alignment as well. Mandelker et al. [17] defined problematic regions for GRCh37 exome which were often used in clinical testing. Methods in this class implicitly model biases in input reads and alignment algorithms. This helps variant calling given reads and alignment algorithms of similar property but is biased against new data types and alternative aligners. These methods also demand more computing and are harder to reproduce.

In this article, we will describe a new method to produce easy regions from multiple high-quality human assemblies [18]. It considers non-reference samples like the second class of methods but uses principles in the first class. It is also not biased towards sequencing data types or existing alignment algorithms.

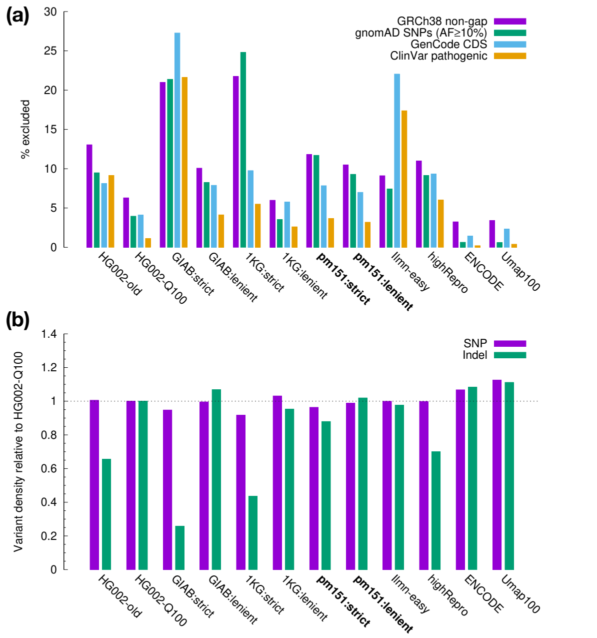

Figure 1. Properties of easy and confident regions. **(a)** Coverage on GRCh38 chromosomal sequences. The gnomAD v4.1 coverage is calculated from SNPs of allele frequency ≥10% intersected with HPRC SNPs. CDS coverage is measured with MANE-selected protein coding genes from GenCode v48. ClinVar coverage is measured on pathogenic and likely pathogenic variants from ClinVar 20250623. **(b)** Variant density relative to HG002-Q100. The variant density of an easy region set is the number of HG002-Q100 variant calls in the easy regions divided by the total region length. The relative density equals to the density of the set over the density in HG002-Q100 confident regions.

Methods

Suppose there are N genomes including the reference genome. A k-mer is *sufficiently unique* if 1) it has only one hit of edit distance up to d_1 in the reference genome and 2) it has $< c \cdot N$ hits of edit distance up to d_2 across all genomes, $d_2 > d_1$. $c > 1$ corresponds to the tolerance with copy-number gains. With the first condition, the k-mer is unlikely to be mismapped under sequencing errors or mutations if the second best hit d_1-edit away. The second condition kicks in when the k-mer is unique in the reference genome but has extra paralogous copies in non-reference samples. In this case, the paralogous copies would be wrongly mapped to the unique reference position and differences between paralogous copies would be called as false variants. The second condition disregards the case where a k-mer is deleted in some genomes but duplicated in others. This is a weakness of our method.

We could test the sufficient uniqueness of a reference k-mer by aligning it each genome separately. This would be computationally intensive. Instead, we construct a ropebwt3 index for all genomes including the reference [19] and align a k-mer with the revised BWA-SW algorithm in the end-to-end mode. This algorithm guarantees to find all exact matches and also reports suboptimal hits, though it may miss inexact hits due to heuristics. We sample one k-mer per w bases from the reference genome. If a k-mer at position i is not sufficiently unique, we mask interval $[i, i + k)$. After processing all sampled k-mers, the remaining regions constitute the easy regions.

On our dataset, $N = 472$. We use $k = 151$ as 151bp reads are common nowadays. We set $w = 10$, $c = 1.01$, $d_1 = 3$ and $d_2 = 7$. Increasing c to 1.02 adds 0.2% of regions; increasing d_2 to 8 reduces regions by 0.1%. During the development of panmask, we additionally generated regions for $N = 100$ and $N = 398$ under a few other settings. We found reasonable parameters generally resulted in similar regions with slightly different balance points between region size and easiness. We also expect our current setting applicable to other species.

To save missed hits due to k-mer sampling and alignment heuristics, we additionally run GenMap [10], an exact algorithm though not supporting gapped alignment, with $k = 151$ and maximal hamming distance 3 on the reference genome. We drop a k-mer region if GenMap finds multiple hits. We also filter out an easy region shorter than 50bp to reduce fragmentation.

We call easy regions produced above as `pm151:lenient`, where "pm" stands for panmask. As SNP and indel calling errors are enriched in low-complexity regions (LCRs; 20), we further exclude such regions longer than 18bp with the SDUST algorithm [21]. This results in `pm151:strict` easy regions which are a strict subset of `pm151:lenient`.

Results

We collected the following easy regions: updated `1KG` [12], `Umap100` for 100bp single-end reads [9], `ENCODE` blacklist v2 [16], reproduced Illumina regions (labeled as `Ilmn-easy`; 13), highly reproducible regions (labeled as `highRepro`; 15) and `GIAB` genome stratification [11]. Both `1KG` and `GIAB` provide relaxed and strict versions. To the best of our knowledge, these are all the available easy regions on GRCh38. We additionally included confident regions `HG002-old` from GIAB ground truth v4.2.1 and `HG002-Q100` from GIAB's new Q100 benchmark v1.1.

We calculated the coverage of each set of regions (Fig. 1a). ENCODE and Umap100 exclude <4% of genomic regions and are the most inclusive; GIAB:strict and 1KG:strict exclude >20% of genomic regions and are the smallest. Our pm151 sets are something in between. Most region sets include higher percentage of coding regions (CDS) than whole genome, which would be expected as CDS tends to be less repetitive. Interestingly, Ilmn-easy misses >20% of CDS

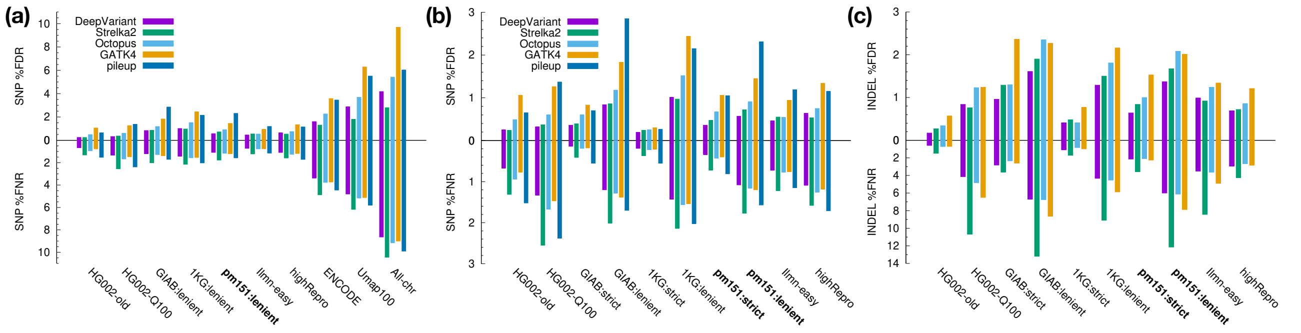

Figure 2. Small variant calling accuracy in easy and confident regions. **(a)** SNP accuracy. DeepVariant, Strelka2, Octopus and GATK short-read variant calls from 30X NovaSeq data were obtained from Baid et al. [25]. Pipelup calls were made with "`minipileup -ys3 -a1 -p.25`". False discovery rate (FDR) and false negative rate (FNR) were calculated by RTG vcfeval [26] with genotype errors ignored. "All-chr" represents all chromosomal sequences in GRCh38 without easy regions. **(b)** SNP accuracy in high-quality easy regions. **(c)** Indel accuracy in high-quality easy regions. Pileup indel calls are omitted due to their high error rates.

albeit its high genome-wide coverage. We suspect this is caused by the GC bias in short reads. The Ilmn-easy regions were derived from the alignment of Illumina short reads. Regions with read depth lower than 75% of the genome average were excluded. However, due to the sequencing bias against high-GC regions [22] and the GC-rich nature of human CDS, CDS would tend to have lower coverage and get excluded. The 1KG easy regions were constructed in a similar way but with a read-depth threshold of 50% instead of 75%. They are less affected by the coverage bias. GIAB:strict excludes regions of extreme GC and is also biased against CDS.

To measure the coverage of SNP catalogs, we acquired SNPs of allele frequency of ≥10% from gnomAD v4.1 [23]. There are 5.85 million SNPs that have passed the gnomAD filters. 96.9% of them are also called from 462 HPRC assemblies. They are likely to be correct. pm151 missed ~10% of these common SNPs (Fig. 1a). This suggests that taking full advantage of input data, sophisticated SNP calling pipelines still have more power over easy regions. On the other hand, we see a sharp difference in the fraction of gnomAD-filtered SNPs: only 1.2% of candidate SNPs inside pm151:strict are filtered by gnomAD, but the percentage rises to 16.6% for candidate SNPs outside pm151:strict. gnomAD also thinks SNPs outside easy regions are harder to call.

We next compared the SNP and indel density using the GIAB Q100 callset. This callset was generated from the near complete assembly of HG002 [24]. It contains variant calls across the whole genome. Although calls outside the HG002-Q100 confident regions are less reliable, most of these calls should also be outside easy regions and would not greatly affect the density analysis. We note that while the SNP density is comparable across easy regions, the indel density varies greatly (Fig. 1b). This is driven by the content of short tandem repeats (STRs) in easy regions as STRs harbor most of indels. GIAB:strict explicitly filters out STRs and has the lowest indel density. pm151:strict filters long LCRs and has reduced density as well. The lower indel density in 1KG:strict might be related to its stringent mapping quality filtering as BWA-MEM, the algorithm used for alignment, tends to give low mapping quality in long LCRs.

To measure the ease of calling, we compared the short-read variant calls by Baid et al. [25] to the GIAB Q100 callset Importantly, although Q100 calls could be wrong outside the HG002-Q100 confident regions, they are still more accurate than short-read calls and even when Q100 calls are wrong, their consistency with short-read calls still reflects the "easiness" of easy regions.

We can see that the SNP calling error rates reach ~10% when no easy regions are applied ("All-chr" in Fig. 2a) but are reduced by several folds to a couple of percent in GIAB, 1KG, pm151 and Ilmn-easy regions. This sharp contrast manifests the necessity of easy regions. Umap100 only considers exact 100-mer matches and overlooks the fact that high-identity inexact matches are still challenging for aligners to distinguish. It is inadequate as easy regions. ENCODE blacklist is better than Umap100 but is still noticeably harder than other easy regions.

1KG:strict consists of the easiest regions (Fig. 2b and 2c). It is almost a subset of pm151:lenient with 99.3% included in the latter. This overlap is higher than between 1KG:strict and Ilmn-easy (97.3%) or GIAB:lenient (98.6%). Excluding long LCRs, pm151:strict greatly reduces SNP and indel calling errors in comparison to pm151:lenient and achieves a reasonable balance between coverage and easiness.

Discussions

The pm151 easy regions are used for filtering spurious variant calls in centromeres, long repeats, or other genomic regions where short-read mapping is likely problematic. These easy regions are not biased towards existing short-read data or aligners in use. Given a prebuilt ropebwt3 index, they can be generated in two days for an arbitrary human assembly on a server with 64 CPU threads, with most time spent on ropebwt3 alignment. The procedure can also be applied to a species with multiple well assembled genomes.

Overall, no easy region set is better than the others on all metrics. Nevertheless, for the purpose of variant filtering, we do not recommend GIAB:strict and Ilmn-easy as they miss ~20% of CDS and ClinVar pathogenic variants; we discourage the use of ENCODE and Umap100 as they are too permissive and are not effective for variant filtering. 1KG and pm151 are the better choices though the 1KG set is only available to GRCh38.

We also caution that although easy regions provide a convenient way to identify high-quality variant calls, they are conservative due to their generality. They do not fully replace other variant filtering strategies. Sophisticated variant calling pipelines optimized for input data and the best aligners and callers can achieve higher power and call more SNPs to high accuracy.

Data availability

pm151 easy regions can be downloaded from Zenodo [27]. Scripts to generate easy regions are available at GitHub [28]. A version of record snapshot of the GitHub repository has been archived in the Software Heritage with the PID swh:1:snp:73333c47e83849c9dec4ff7f26cd41c413872a1d [29]. All data used in this project are available in the GigaScience database, GigaDB [30].

Competing Interests

The author declares he has no competing interests.

Funding

This work is supported by National Institute of Health grant R01HG010040, U01HG013748 and U41HG010972 (to H.L.).

Author's Contributions

H.L. conceived the project, implemented the method, analyzed the data and drafted the manuscript.

Acknowledgements

We are grateful to Arang Rhie and Yoo-Jin Ha for pointing us to easy regions derived by Illumina and the 1000 Genomics Project, respectively. We would like to acknowledge the National Genome Research Institute (NHGRI) for funding the following grants supporting the creation of the human pangenome reference: U41HG010972, U01HG010971, U01HG013760, U01HG013755, U01HG013748, U01HG013744, R01HG011274, and the Human Pangenome Reference Consortium (BioProject ID: PRJNA730823).

References

1. Depristo MA, Banks E, Poplin R, Garimella KV, Maguire JR, et al. A framework for variation discovery and genotyping using next-generation DNA sequencing data. Nat Genet 2011;43:491–8.
2. Kim S, Scheffler K, Halpern AL, Bekritsky MA, Noh E, Källberg M, et al. Strelka2: fast and accurate calling of germline and somatic variants. Nat Methods 2018;15:591–594.
3. Poplin R, Chang PC, Alexander D, Schwartz S, Colthurst T, et al. A universal SNP and small-indel variant caller using deep neural networks. Nat Biotechnol 2018;36:983–987.
4. Cooke DP, Wedge DC, Lunter G. A unified haplotype-based method for accurate and comprehensive variant calling. Nat Biotechnol 2021;39:885–892.
5. Zook JM, Chapman B, Wang J, Mittelman D, Hofmann O, et al. Integrating human sequence data sets provides a resource of benchmark SNP and indel genotype calls. Nat Biotechnol 2014;32:246–51.
6. Li H, Bloom JM, Farjoun Y, Fleharty M, Gauthier L, et al. A synthetic-diploid benchmark for accurate variant-calling evaluation. Nat Methods 2018;15:595–597.
7. Olson ND, Wagner J, McDaniel J, Stephens SH, Westreich ST, et al. PrecisionFDA Truth Challenge V2: Calling variants from short and long reads in difficult-to-map regions. Cell Genom 2022;2:100129.
8. Marco-Sola S, Sammeth M, Guigó R, Ribeca P. The GEM mapper: fast, accurate and versatile alignment by filtration. Nat Methods 2012;9:1185–8.
9. Karimzadeh M, Ernst C, Kundaje A, Hoffman MM. Umap and Bismap: quantifying genome and methylome mappability. Nucleic Acids Res 2018;46:e120.
10. Pockrandt C, Alzamel M, Iliopoulos CS, Reinert K. GenMap: ultra-fast computation of genome mappability. Bioinformatics 2020;36:3687–3692.
11. Dwarshuis N, Kalra D, McDaniel J, Sanio P, Alvarez Jerez P, et al. The GIAB genomic stratifications resource for human reference genomes. Nat Commun 2024;15:9029.
12. 1000 Genomes Project Consortium. A map of human genome variation from population-scale sequencing. Nature 2010;467:1061–73.
13. Aganezov S, Yan SM, Soto DC, Kirsche M, Zarate S, et al. A complete reference genome improves analysis of human genetic variation. Science 2022;376:eabl3533.
14. Nurk S, Koren S, Rhie A, Rautiainen M, Bzikadze AV, et al. The complete sequence of a human genome. Science 2022;376:44–53.
15. Pan B, Ren L, Onuchic V, Guan M, Kusko R, et al. Assessing reproducibility of inherited variants detected with short-read whole genome sequencing. Genome Biol 2022;23:2.
16. Amemiya HM, Kundaje A, Boyle AP. The ENCODE Blacklist: Identification of Problematic Regions of the Genome. Sci Rep 2019;9:9354.
17. Mandelker D, Schmidt RJ, Ankala A, McDonald Gibson K, Bowser M, et al. Navigating highly homologous genes in a molecular diagnostic setting: a resource for clinical next-generation sequencing. Genet Med 2016;18:1282–1289.
18. Liao WW, Asri M, Ebler J, Doerr D, Haukness M, et al. A draft human pangenome reference. Nature 2023;617:312–324.
19. Li H. BWT construction and search at the terabase scale. Bioinformatics 2024;40:btae717.
20. Li H. Toward better understanding of artifacts in variant calling from high-coverage samples. Bioinformatics 2014;30:2843–2851.
21. Morgulis A, Gertz EM, Schäffer AA, Agarwala R. A fast and symmetric DUST implementation to mask low-complexity DNA sequences. J Comput Biol 2006;13:1028–40.
22. Benjamini Y, Speed TP. Summarizing and correcting the GC content bias in high-throughput sequencing. Nucleic Acids Res 2012;40:e72.
23. Karczewski KJ, Francioli LC, Tiao G, Cummings BB, Alföldi J, et al. The mutational constraint spectrum quantified from variation in 141,456 humans. Nature 2020;581:434–443.
24. Rautiainen M, Nurk S, Walenz BP, Logsdon GA, Porubsky D, et al. Telomere-to-telomere assembly of diploid chromosomes with Verkko. Nat Biotechnol 2023;41:1474–1482.
25. Baid G, Nattestad M, Kolesnikov A, Goel S, Yang H, Chang PC, et al. An Extensive Sequence Dataset of Gold-Standard Samples for Benchmarking and Development. bioRxiv 2020;.
26. Cleary JG, Braithwaite R, Gaastra K, Hilbush BS, Inglis S, et al. Comparing Variant Call Files for Performance Benchmarking of Next-Generation Sequencing Variant Calling Pipelines. bioRxiv 2015;.
27. H Li. Easy genomic regions for short-read variant calling. Zenodo. DOI 10.5281/zenodo.14903542.
28. Li H. panmask. https://github.com/lh3/panmask. [Computer software]. Accessed 26 July 2025.
29. Li H. panmask. [Software Heritage Archive]. 2025. https://archive.softwareheritage.org/swh:1:snp:73333c47e83849c9dec4ff7f26cd41c413872a1d;origin=https://github.com/lh3/panmask. Accessed 26 July 2025.
30. Li H. Supporting data for "Finding easy regions for short-read variant calling from pangenome data" GigaScience Database. 2025. https://doi.org/10.5524/102750

Your PDF file "panmask.pdf" cannot be opened and processed. Please see the common list of problems, and suggested resolutions below.

Reason:

Other Common Problems When Creating a PDF from a PDF file

You will need to convert your PDF file to another format or fix the current PDF file, then re-submit it.

A1.0.1: In addition to the responses to reviewers' comments, I made two other minor improvements to the manuscript:

1. I missed the "highRepro" easy region set from Pan et al (2022). I have added it to the figures. This does not change the overall conclusion.
2. I developed a new algorithm, longdust, to identify low-complexity regions and found the new regions are overall better (more inclusive, easier, and less fragmented) than the old SDUST regions. I have updated pm151:strict accordingly. pm151:lenient stays the same.

Reviewer #1: The author describes a pangenome-based approach to produce sample-agnostic easy regions for filtering potentially incorrect variant calls in human samples. Data and code are both available.

Q1.1.1: I just have questions about the parameters used for the human pangenome and how to set them in general. "On our dataset, N = 472 and we set k = 151, w = 10, d1 = 3 and d2 = 7." Why did the author choose these parameters? Which metric did he optimize to find them? Would it make sense to modify those parameters in light of the analysis to be conducted with the human data? How should researchers set these parameters for other pangenomes (primates, plants, bacteria, etc...)?

A1.1.1: I have added a new paragraph to explain alternative settings I have tried:

"*On our dataset, N = 472. We use k = 151 as 151bp reads are common nowadays. We set w = 10, c = 1.01, d1 = 3 and d2 = 7. Increasing c to 1.02 adds 0.2% of regions; increasing d2 to 8 reduces regions by 0.1%. During the development of panmask, we additionally generated regions for N = 100 and N = 398 under a few other settings. We found reasonable parameters generally resulted in similar regions with slightly different balance points between region size and easiness. We also expect our current setting applicable to other species.*"

Reviewer #2: This paper addresses the question "how does one know which parts of a given genome can be analyzed confidently with short-read sequencing, mapping, and variant calling"? For human genome builds GRCh37 and GRCh38, confident regions have been determined by NIST GIAB with great expertise and effort. This manuscript proposes a set of clearly defined and runnable heuristics to empirically define a set of confident regions for a given sample or panel of samples. This is useful for population studies to understand whether there are different genomics regions that are more difficult to analyze in that population, and potentially for non-human species which lack confident regions generated for any individual.

The paper proposes a mapping-based approach from kmers, centered around a threshold for tolerable mismatches. This approach is reasonable and has the advantage of being clearly defined and easy to run. The paper then evaluates the error rates of several standard methods with confident regions generated by the approach against the documented error rates from various gold standard and exhaustively determined truth sets. Overall, my interpretation is that the error rates observed via the proposed method are generally consistent with the stringency seen in GIAB T2T confident regions. Meaning that in conclusion this is a method that can create

similar stringency regions to T2T in a sample on demand.

The methods are reasonable and clearly defined. The paper is very succinctly and clearly written, the figures are easy to interpret, and the findings are clear from the results. I feel the work can be published as-is. I have some observations and comments, but they just observations, not requests for any edits:

Additional observations:

Q1.2.1: First: The variant callers themselves are also useful and important to understanding the reliability of a call. Specifically, the Genotype Quality (GQ) field (hopefully) contains well-calibrated probabilities that a site is genotyped correctly, while the QUAL field (hopefully) contains well-calibrated probabilities about whether a variant exists. Ideally, these accurately reflect empirical error rates, and are used to help understand reliability of a call. It would be interesting to understand how variant probabilities correlate with empirical error within outside different confident regions. It may be the case that in some places outside a confident region, the variant caller can give confident and correct outputs, in others it may correctly say it is unconfident, and in others indicate incorrectly that it is confident in an erroneous call.

Producing confident regions in the manner of the paper is useful as an orthogonal annotation, but the variant confidence is hopefully another useful signal to understand confidence as well.

A1.2.1: Easy regions exclude regions prone to mapping artifacts. How QUAL is affected depends on whether QUAL considers these artifacts. GATK outputs high QUAL even given a false SNP in CNVs. Its QUAL is not effective against mapping artifacts. GATK has VQSR but it should not be applied to a single sample (though many researchers are doing that). Trained from data, DeepVariant outputs reasonable QUAL that takes mapping artifacts into account.

That said, the average QUAL is indeed lower outside easy regions across all callers: GATK: 759.902→727.016, Strelka2: 252.106→218.689, Octopus: 649.204→531.897 and DeepVariant: 38.5063→28.5465. We see a larger relative drop for DeepVariant probably because its estimate is closer to the empirical probability. I did not discuss these in the manuscript as the discussion is caller specific and hard to interpret for general audience.

Q1.2.2: Second: This resource is useful as annotations to understand which variants might need a higher standard of evidence, or more inspection (either computationally or wet lab validation), but I hope readers will not conclude that variants should not be looked at outside these regions. Ultimately, it will depend on the downstream task that a user is conducting and their tolerance for missing a variant versus having to manage false positives. The paper does not suggest otherwise, and does not need correction in this respect. I am just thinking about some of the different ways people could read the work.

A1.2.2: I fully agree. In the revision, I have added a comparison to gnomAD common SNPs to show that easy regions may miss SNPs callable by gnomAD.

“*To measure the coverage of SNP catalogs, we acquired SNPs of allele frequency of ⩾10% from gnomAD v4.1 (Karczewski et al., 2020). There are 5.85 million SNPs that have passed the gnomAD filters. 96.9% of them are also called from 462 HPRC assemblies. They are likely to be correct. pm151 missed~10% of these common SNPs (Fig. 1a). This suggests that taking full advantage of input data, sophisticated SNP calling pipelines still have more power over easy regions. On the other hand, we see a sharp difference in the fraction of gnomAD-filtered SNPs: only 1.2% of candidate SNPs inside pm151:strict are filtered by gnomAD, but the percentage rises to 16.6% for candidate SNPs outside pm151:strict. gnomAD also thinks SNPs outside easy regions are harder to call.*”

To emphasize the point further, I now conclude the manuscript with:

“*We also caution that although easy regions provide a convenient way to identify high-quality variant calls, they are conservative due to their generality. They do not fully replace other variant filtering strategies. Sophisticated variant calling pipelines optimized for input data and the best aligners and callers can achieve higher power and call more SNPs to high accuracy.*”

Q1.2.3: Third: I see genotype errors set to be ignored in the evaluation by RTGeval. It's generally my instinct to include genotype errors, as it is important to determine HET versus HOM correctly. I think I understand why the author would eval like this. Plotting results as FDR versus FNR is a very compact and visually interpretable representation. A genotype error is a different category, or you have to decide if it makes sense to double count as both a FDR+FNR for the plot. I don't expect it would change any conclusion to include GT errors, so this is only a difference in choice, not any issue.

A1.2.3: As the reviewer correctly pointed out, rtgeval would count a genotype error both as a false positive and as a false negative. This is counterintuitive in that a genotype error is minor relative to a false SNP allele but is penalized more heavily. I think this is a questionable decision, so I wrote a script to separate genotype errors from allelic errors based on the rtgeval raw output (Li, 2014). In general, though, allelic errors, genotype errors and direct rtgeval-reported errors are similar and are highly correlated. This would not change the conclusion of the manuscript, so I kept the measurement the same as the initial version.

Q1.2.4: To the extent I have a suggestion about figure 2, it would be to consider bolding the pm151 labels in the x-axis. This can make it faster for a reader to realize that the relevant comparison to make is between that category and the others to conclude which confident regions the method here is similar to.

A1.2.4: Done. I thank the reviewer for the suggestion.
