# Supplementary material for: Finding easy regions for short-read variant calling from pangenome data
Source: Gigascience. 2025 Sep 4;14:giaf103. doi: 10.1093/gigascience/giaf103 (PMC12409275; doi:10.1093/gigascience/giaf103)
Supplement: giaf103_Reviewer_2_Report_Original_Submission — Andrew Carroll -- 8/2/2025 [file giaf103_reviewer_2_report_original_submission.pdf]

Reviewer Report

Title: Finding easy regions for short-read variant calling from pangenome data

Version: Original Submission **Date:** 8/2/2025

Reviewer name: Andrew Carroll

Reviewer Comments to Author:

This paper addresses the question "how does one know which parts of a given genome can be analyzed confidently with short-read sequencing, mapping, and variant calling"? For human genome builds GRCh37 and GRCh38, confident regions have been determined by NIST GIAB with great expertise and effort. This manuscript proposes a set of clearly defined and runnable heuristics to empirically define a set of confident regions for a given sample or panel of samples. This is useful for population studies to understand whether there are different genomics regions that are more difficult to analyze in that population, and potentially for non-human species which lack confident regions generated for any individual.The paper proposes a mapping-based approach from kmers, centered around a threshold for tolerable mismatches. This approach is reasonable and has the advantage of being clearly defined and easy to run. The paper then evaluates the error rates of several standard methods with confident regions generated by the approach against the documented error rates from various gold standard and exhaustively determined truth sets. Overall, my interpretation is that the error rates observed via the proposed method are generally consistent with the stringency seen in GIAB T2T confident regions. Meaning that in conclusion this is a method that can create similar stringency regions to T2T in a sample on demand.The methods are reasonable and clearly defined. The paper is very succinctly and clearly written, the figures are easy to interpret, and the findings are clear from the results. I feel the work can be published as-is. I have some observations and comments, but they just observations, not requests for any edits:Additional observations:First:The variant callers themselves are also useful and important to understanding the reliability of a call. Specifically, the Genotype Quality (GQ) field (hopefully) contains well-calibrated probabilities that a site is genotyped correctly, while the QUAL field (hopefully) contains well-calibrated probabilities about whether a variant exists. Ideally, these accurately reflect empirical error rates, and are used to help understand reliability of a call. It would be interesting to understand how variant probabilities correlate with empirical error within outside different confident regions. It may be the case that in some places outside a confident region, the variant caller can give confident and correct outputs, in others it may correctly say it is unconfident, and in others indicate incorrectly that it is confident in an erroneous call.Producing confident regions in the manner of the paper is useful as an orthogonal annotation, but the variant confidence is hopefully another useful signal to understand confidence as well.Second:This resource is useful as annotations to understand which variants might need a higher standard of evidence, or more inspection (either computationally or wet lab validation), but I hope readers will not conclude that variants should not be looked at outside these regions. Ultimately, it will depend on the downstream task that a user is conducting and their tolerance for missing a variant versus having to manage false positives. The paper does not suggest otherwise, and does not need correction in this respect. I am just thinking about some of the different ways people

could read the work.Third:I see genotype errors set to be ignored in the evaluation by RTGeval. It's generally my instinct to include genotype errors, as it is important to determine HET versus HOM correctly. I think I understand why the author would eval like this. Plotting results as FDR versus FNR is a very compact and visually interpretable representation. A genotype error is a different category, or you have to decide if it makes sense to double count as both a FDR+FNR for the plot. I don't expect it would change any conclusion to include GT errors, so this is only a difference in choice, not any issue.To the extent I have a suggestion about figure 2, it would be to consider bolding the pm151 labels in the x-axis. This can make it faster for a reader to realize that the relevant comparison to make is between that category and the others to conclude which confident regions the method here is similar to.

Level of Interest

Please indicate how interesting you found the manuscript: Choose an item.

Quality of Written English

Please indicate the quality of language in the manuscript: Choose an item.

Declaration of Competing Interests

Please complete a declaration of competing interests, considering the following questions:

- Have you in the past five years received reimbursements, fees, funding, or salary from an organisation that may in any way gain or lose financially from the publication of this manuscript, either now or in the future?
- Do you hold any stocks or shares in an organisation that may in any way gain or lose financially from the publication of this manuscript, either now or in the future?
- Do you hold or are you currently applying for any patents relating to the content of the manuscript?
- Have you received reimbursements, fees, funding, or salary from an organization that holds or has applied for patents relating to the content of the manuscript?
- Do you have any other financial competing interests?
- Do you have any non-financial competing interests in relation to this paper?

If you can answer no to all of the above, write 'I declare that I have no competing interests' below. If your reply is yes to any, please give details below.

I declare that I have no competing interests.

I agree to the open peer review policy of the journal. I understand that my name will be included on my report to the authors and, if the manuscript is accepted for publication, my named report including any attachments I upload will be posted on the website along with the authors' responses. I agree for my

report to be made available under an Open Access Creative Commons CC-BY license (http://creativecommons.org/licenses/by/4.0/). I understand that any comments which I do not wish to be included in my named report can be included as confidential comments to the editors, which will not be published.

Choose an item.

To further support our reviewers, we have joined with Publons, where you can gain additional credit to further highlight your hard work (see: https://publons.com/journal/530/gigascience). On publication of this paper, your review will be automatically added to Publons, you can then choose whether or not to claim your Publons credit. I understand this statement.

Yes Choose an item.
